# Evaluation of photobiomodulation therapy for genitourinary syndrome of menopause: A single-center prospective study

**DOI:** 10.1371/journal.pone.0351765

**Published:** 2026-06-22

**Authors:** Pei-Chi Wu, Ho-Hsiung Lin, Chi-Hau Chen, Yih-Shing Kuo

**Affiliations:** 1 Department of Obstetrics and Gynecology, National Taiwan University Hospital, Taipei, Taiwan; 2 Department of Obstetrics and Gynecology, National Taiwan University Hospital Bei-Hu Branch, Taipei, Taiwan; 3 Department of Obstetrics and Gynecology, Far Eastern Memorial Hospital, New Taipei, Taiwan; Massachusetts General Hospital, UNITED STATES OF AMERICA

## Abstract

**Background:**

Genitourinary syndrome of menopause (GSM) is common, and non-hormonal treatment options are needed for women unsuitable for or reluctant to use estrogen therapy.

**Objective:**

To prospectively evaluate clinical outcomes and clinical improvement rates of photobiomodulation (PBM) for GSM.

**Methods:**

Twenty-seven postmenopausal women with GSM were enrolled in a prospective study conducted from January 2024 to July 2025. Participants received eight weekly 30-minute sessions of pulsed 660-nm PBM delivered through a stationary silicone vaginal light-guide probe. With the probe attached, target irradiance was 32.38mW/cm² at the probe tip and 13.38mW/cm² at the probe side, corresponding to per-session radiant exposures of 29.14J/cm² at the vaginal fornix and 12.04J/cm² at the vaginal wall. Assessments occurred at baseline (V1), pre-4th session (V2), pre-8th session (V3), and three months post-treatment (V4). The primary outcome was the Vaginal Health Index (VHI). Secondary outcomes included validated questionnaires for lower urinary tract symptoms (LUTS) and sexual function (FSFI). Clinical improvement was defined using established minimal clinically important differences (MCID), minimal detectable change (MDC), and cutoff scores as thresholds.

**Results:**

The mean age of participants was 62.9 ± 9.7 years. Total VHI scores were significantly higher at all follow-up time points compared to baseline (*p* < 0.05), with all participants reaching the non-atrophic VHI threshold at V4. UDI-6, IIQ-7, OABSS, and several KHQ domains improved significantly (*p* < 0.05) and persisted through the 3-month follow-up. Total FSFI scores improved significantly (p < 0.05), but only 13.3% reached the clinical cutoff (≥26.5) at V4 for functional recovery. No adverse events were observed during the study period.

**Conclusions:**

Intravaginal 660-nm PBM is a well-tolerated, non-hormonal modality that may improve objective vaginal health and subjective symptoms in GSM. As an integrated therapeutic option, PBM may also improve GSM-related LUTS, suggesting potential relevance for geriatric patients with polypharmacy concerns.

**Trial registration:**

ClinicalTrials.gov NCT06074120

## Introduction

Genitourinary syndrome of menopause (GSM) represents a significant clinical challenge, encompassing a spectrum of symptoms such as vaginal dryness, irritation, dyspareunia, and various lower urinary tract symptoms (LUTS) [1,2]. These symptoms profoundly impact the quality of life and sexual health of postmenopausal women [3]. While topical estrogens remain the primary treatments, their use is often restricted by contraindications—particularly in survivors of hormone-dependent cancers—or limited by patient hesitancy and poor long-term compliance [4]. Consequently, there is an increasing demand for effective, non-hormonal, and minimally invasive therapeutic alternatives.

Over the past decade, vaginal laser therapy has emerged as a promising modality. By stimulating collagen synthesis and enhancing microcirculation, laser therapy facilitates the regeneration of the vaginal mucosa [5,6]. Among the available technologies, photobiomodulation (PBM), historically referred to as low-level laser therapy (LLLT), offers a distinct advantage as a “cold laser” treatment [7–9]. Unlike ablative high-energy lasers, PBM initiates biochemical reactions at the cellular level to promote tissue healing and reduce inflammation without inducing thermal injury or procedural pain [10].

A previous pilot study explored the preliminary efficacy of PBM for both GSM and stress urinary incontinence (SUI) [11]. The findings indicated that while both conditions showed improvement, the therapeutic response was substantially more pronounced and consistent in patients with GSM compared to those with SUI [11]. Furthermore, the procedure demonstrated an excellent safety profile, with no adverse effects or procedural discomfort observed, likely due to the non-ablative nature of the static silicone vaginal probe.

Given these preliminary findings and the high patient tolerability observed in clinical practice, especially for women with GSM, further validation through a prospective approach is warranted, as prospective evidence quantifying the specific improvement rates across multiple dimensions remains lacking. Therefore, this study primarily aimed to evaluate the efficacy of PBM for GSM through serial VHI assessment, while secondarily establishing clinical improvement rates for subjective quality-of-life parameters.

## Materials and methods

### Study design and participant enrollment

This prospective study was conducted between January 2024 and July 2025. During this period, all consecutive patients seeking treatment for GSM at our gynecologic outpatient clinic were invited to participate. Patients were fully informed about the study’s objectives and procedures; those who expressed willingness provided written informed consent prior to enrollment for treatment and subsequent analysis. The start and end of the recruitment period for this study were January 12^th^, 2024 and March 7^th^ 2025. The study was approved by the Research Ethics Committee of National Taiwan University Hospital (IRB No. 202307193DIND) and registered at ClinicalTrials.gov (NCT06074120). This prospective study was conducted in accordance with the Strengthening the Reporting of Observational Studies in Epidemiology (STROBE) statement. (S1 File)

### Inclusion and exclusion criteria

The study included postmenopausal women (defined as no spontaneous menstruation for at least one year) diagnosed with GSM. Diagnosis was based on the presence of vulvovaginal dryness, irritation, or dyspareunia, as well as LUTS, including storage and/or voiding symptoms. Exclusion criteria consisted of: (1) ≥stage 2 pelvic organ prolapse according to the pelvic organ prolapse quantification system; (2) active urinary tract infection or pelvic infection within the previous two weeks; (3) preexisting pelvic malignancies; and (4) abnormal sensory nerve or coagulation.

### Photobiomodulation protocol and serial assessments

**Fig 1:**
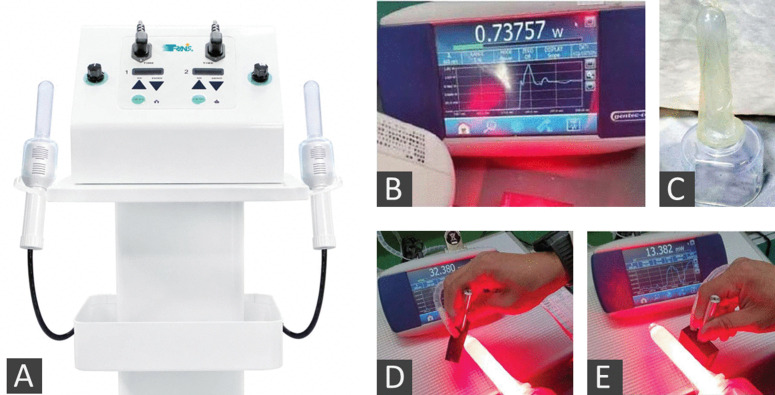
Photobiomodulation (PBM) therapy system and the vaginal probe for genitourinary syndrome of menopause. The system utilizes an AlGaInP semiconductor diode laser with a center wavelength of 660 nm, operated in pulsed mode to deliver uniform, circumferential irradiation. The stationary contact design ensures consistent and targeted energy delivery to the entire vaginal mucosal surface throughout the 30-minute treatment session. **(A)** The TI-816-12 “TRANS” Laser Light Therapy Apparatus (Transverse Industries Co., Ltd, New Taipei, Taiwan). **(B)** Representative monitor display of the peak radiant power measurement (737.57 mW). **(C)** Clinical preparation showing the silicone light-guide vaginal probe protected by a prophylactic cover. **(D)** Measurement of the target irradiance at the probe tip (32.380 mW/cm^2^). **(E)** Measurement of the target irradiance at the probe side (13.382 mW/cm^2^).

Regarding the PBM protocol, the treatment was administered once a week for eight consecutive weeks [11]. The technical and radiometric parameters of PBM are shown in Table 1 in accordance with the guideline [12]. The AlGaInP semiconductor diode laser system (TI-816–12, Transverse Industries Co., Ltd, New Taipei, Taiwan), and its silicone light-guide vaginal probe are illustrated in Fig 1. During the procedure, patients assumed a supine position. A stationary contact technique was utilized, in which the physician inserted the probe to the posterior fornix. This configuration ensured uniform, circumferential irradiation of the entire vaginal mucosal surface for a 30-minute session in pulsed mode. Post-treatment education included abstaining from sexual activity, baths, or hot springs for 48 hours, and refraining from vaginal douching.

**Table 1:** Technical specifications and radiometric parameters of the photobiomodulation (PBM) therapy system for genitourinary syndrome of menopause (GSM).

Parameter	Value	Notes/ Rationale
Device Information		
Manufacturer/ Model	Transverse Industries Co., Ltd, Taiwan — TI-816–12	
Emitter type	AlGaInP semiconductor diode laser	
Number of emitters	Single	
Spatial distribution of emitters	Single emitter at probe center	
Beam delivery system	Silicone light guide vaginal probe	
Beam/ Irradiation Parameters		
Center wavelength	660 nm	Measured
Spectral bandwidth	±10nm	Measured
Operating mode	Pulsed	
Frequency	20 Hz	
Pulse on duration	0.025 sec	
Duty cycle	50%	
Energy per pulse	0.0184 J	0.73757Wx0.025s=0.0184J
Polarization	Linear	
Aperture diameter	≤1.2 cm	Measured
Irradiance at aperture	≤646.99 mW/cm^2^	737.57mW ÷ 1.14 cm^2^≒646.99mW/cm^2^
Beam divergence	≈0.35 mrad	Measured
Beam shape	Circular	
Beam profile	Uniform	
Treatment Dosimetry		
Peak radiant power	737.57 mW	Measured
Average radiant power	368.79 mW	737.57mWx0.5 = 368.785mW
Beam spot size at target	≤2 cm^2^	(0.8 cm)^2^π ≒ 2 cm^2^
Irradiance at target	Tip of probe: ≤ 32.38 mW/cm^2^ Side of probe: ≤ 13.38 mW/cm^2^	Measured with probe
Exposure duration	1800 sec (30 min per session)	
Radiant exposure (Fluence)	Vaginal fornix: ≤ 29.14 J/cm^2^ Vaginal wall: ≤ 12.04 J/cm^2^	32.38x1800x0.5÷1000 = 29.14 13.38x1800x0.5÷1000 = 12.04
Radiant energy (per session)	≤841 J	0.03238x2x0.5x1800+ 0.01338x65x0.5x1800≒841J
Number of points irradiated	Whole vaginal mucosa	Contact application
Area irradiated	≈67 cm^2^	2 + 65 = 67 cm^2^
Application technique	Contact	With silicone vaginal probe
Number of sessions	8 sessions	Once per week
Total radiant energy	≤6728 J	841 J/session×8session

At the time of enrollment, a detailed medical history was recorded, and LUTS symptoms were assessed to establish a baseline (V1). All participants underwent serial assessments—each comprising a pelvic examination for vaginal health index (VHI) [13] (vaginal pH was assessed using Macherey-Nagel pH-Fix 3.6–6.1 (Item#: 92130; Macherey-Nagel GmbH & Co. KG, Düren, Germany) evaluation as primary outcome and the completion of all study questionnaires (the International Consultation on Incontinence Questionnaire-Short Form, ICIQ-SF [14]; the Incontinence Impact Questionnaire-7, IIQ-7 [15]; the Urogenital Distress Inventory-6, UDI-6 [15]; the Overactive Bladder Symptoms Score, OABSS [16]; the King’s Health Questionnaire, KHQ [17]; and the Female Sexual Function Index, FSFI [18]) as secondary outcomes—at four distinct time points:

V1: Baseline assessment upon enrollment.

V2: Assessment performed immediately prior to the 4th treatment session.

V3: Assessment performed immediately prior to the 8th treatment session.

V4: Final follow-up assessment conducted three months after the completion of the 8-week treatment course.

To bridge the gap between statistical significance and clinical relevance, clinical improvement was defined by using established minimal clinically important differences (MCID), minimal detectable change (MDC), and cutoff scores as thresholds:

VHI: A cutoff score of >15 defined the restoration of unatrophic vaginal status [19].

FSFI total score: A cutoff of 26.5 was applied to identify the resolution of sexual dysfunction [20].

MCID thresholds were set at 1.7 for ICIQ-SF [21], 3 for OABSS [22], and 5 for KHQ domains [23].

Calculated MDC values of 4.1 for UDI-6 and 3.8 for IIQ-7 (based on raw total scores) were adopted to evaluate meaningful clinical improvements [24].

### Statistical analysis

Statistical analyses were performed using R version 4.3.1 (R Foundation for Statistical Computing, Vienna, Austria). An a priori power analysis was conducted to determine the required sample size; given an estimated effect size of d ≥ 0.6 and an alpha level of 0.05, the final sample size of n = 27 provided a statistical power of approximately 83% to detect significant changes in the primary outcome (VHI total score). Data distribution was formally assessed using the Shapiro-Wilk test. Due to the non-normal distribution of several parameters, continuous variables were summarized as medians with interquartile ranges (IQR). To account for repeated measures across multiple timepoints (V1-V4), we utilized the Friedman test for the initial global comparison of all continuous outcomes. For variables showing statistical significance in the global test, post-hoc pairwise comparisons were conducted using the Holm–Bonferroni correction to control the family-wise Type I error rate. To maintain the integrity of clinical observations, missing values were handled using available-case analysis. Statistical comparisons across timepoints were conducted using all participants who provided valid data for the specific outcome measure. For variables with fluctuating participant numbers, such as the FSFI scores (where n varied based on sexual activity), the analysis was restricted to individuals who provided valid data at each specific timepoint. No statistical imputation was performed. To quantify the magnitude of the therapeutic effect, effect sizes (rank-biserial correlation or Cohen’s d equivalents) and their 95% confidence intervals (CI) were calculated for the primary outcome (VHI). For all analyses, a *p*-value < 0.05 was considered statistically significant.

## Results

### Baseline characteristics

A total of 31 eligible patients were initially assessed for recruitment. Among them, 4 patients declined to participate, and the remaining 27 participants were enrolled, received treatment, and completed scheduled follow-up assessments for final analysis. The mean age of the cohort was 62.9 ± 9.7 years. The mean age at menopause was 49.4 ± 3.5 years, with a mean duration of menopause of 13.4 ± 9.9 years. Regarding parity, only 6 (22%) participants were nulliparous. Most participants presented with varying degrees of LUTS at baseline, with storage symptoms being the most prevalent. Detailed baseline demographics and clinical histories are summarized in Table 2.

**Table 2:** Baseline characteristics of women receiving photobiomodulation (PBM) for genitourinary syndrome of menopause (GSM) (N = 27).

Variables	Value (N = 27)
Demographics	
Age (years), mean ± SD	62.852 ± 9.718
Parity, median (range)	2 (0 - 9)
Nulliparous, n (%)	6 (22.2)
Cesarean Section	0 (0 - 2)
Menopausal History	
Age at menopause(years), mean ± SD	49.4 ± 3.5
Duration of menopause (years), mean ± SD	13.4 ± 9.9
Lower Urinary Tract Symptoms	
Urinary Frequency	18 (66.7%)
Urinary Urgency	19 (70.4%)
Nocturia	13 (48.1%)
Urgency Urinary Incontinence	15 (55.6%)
Stress Urinary Incontinence	10 (37.0%)
Weak Stream	7 (25.9%)
Intermittency	9 (33.3%)
Strain To Void	8 (29.6%)
Incomplete Emptying Sensation	9 (33.3%)
Constipation	11 (40.7%)
Hypertension	6 (22.2%)
Type 2 Diabetes Mellitus	3 (11.1%)

*Note*: Data presented as mean ± standard deviation, median (range), or number (percentage).

### Objective outcomes: Vaginal health index

**Fig 2:**
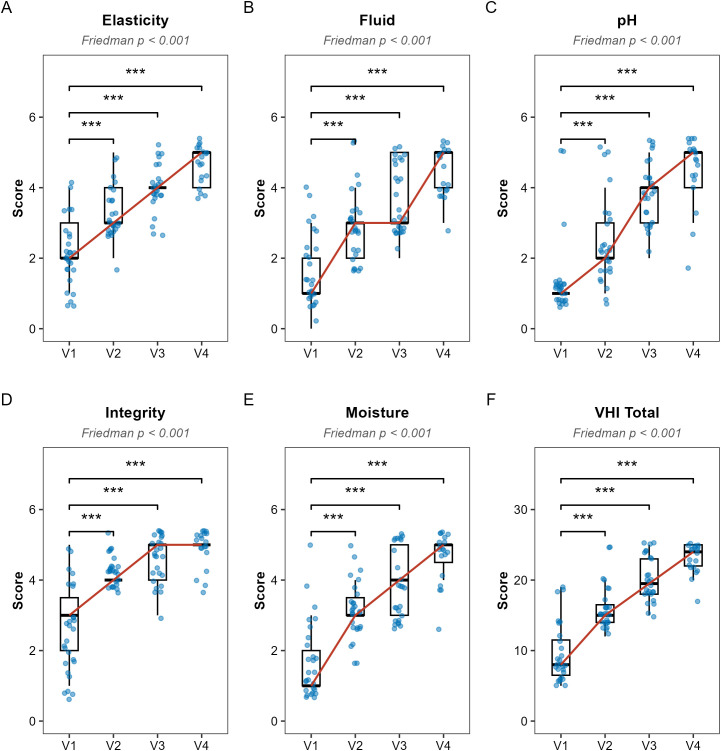
Changes in objective vaginal health parameters assessed by the vaginal health index (VHI) over time. Individual panels show **(A)** Elasticity, **(B)** Fluid, (C) pH, **(D)** Integrity, **(E)** Moisture, and **(F)** VHI Total Score. Data are presented as boxplots (median and interquartile range) overlaid with individual jittered data points. The dashed lines and diamond markers represent the trend of median values. Maximum theoretical scores for each parameter are: 5 for sub-indices, 25 for total score. Significant improvements were observed in all parameters from baseline (V1) to all subsequent follow-up visits (V2–V4). *p < 0.05, **p < 0.01, ***p < 0.001 compared to V1. (The Friedman test was used for global repeated-measures comparisons. When significant, post-hoc pairwise comparisons with baseline were performed using Holm–Bonferroni correction.).

Objective assessment of vaginal health demonstrated comprehensive and significant improvement following PBM (Fig 2). Compared to baseline (V1), total VHI scores increased significantly at all follow-up time points (V2, V3, and V4; all *p* < 0.05). Regarding specific sub-indices, elasticity, fluid, pH, epithelial integrity, and moisture consistently showed statistically significant progress. Notably, these improvements were sustained throughout the period from the initiation of treatment to the final post-treatment follow-up.

### Subjective urinary symptoms and quality of life

**Fig 3:**
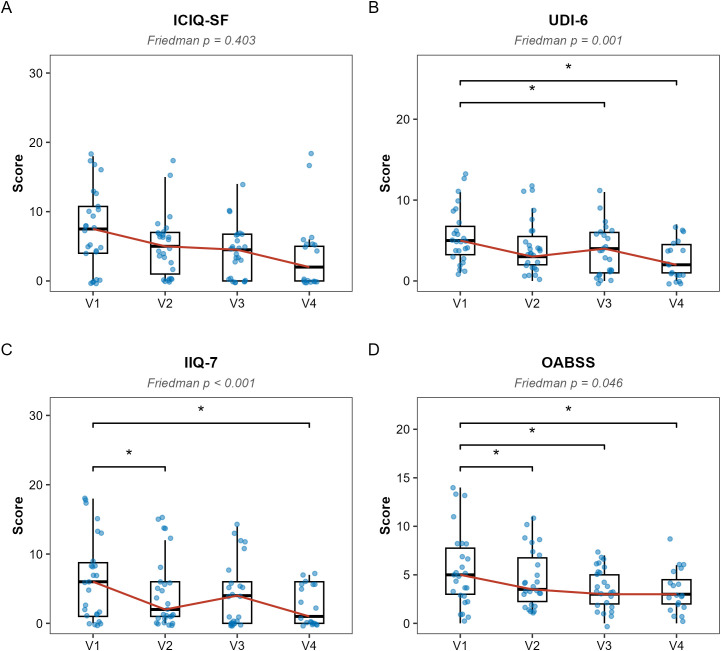
Trends in subjective urinary symptoms and incontinence-related distress scores. **(A)** International Consultation on Incontinence Questionnaire-Short Form (ICIQ-SF), **(B)** Urogenital Distress Inventory-6 (UDI-6), **(C)** Incontinence Impact Questionnaire-7 (IIQ-7), and **(D)** Overactive Bladder Symptom Score (OABSS). Boxplots illustrate the distribution of scores with y-axes adjusted to the maximum possible score of each questionnaire. A downward trend indicates symptom relief. *p < 0.05, **p < 0.01, ***p < 0.001 compared to baseline (V1). (The Friedman test was used for global repeated-measures comparisons. When significant, post-hoc pairwise comparisons with baseline were performed using Holm–Bonferroni correction.).

Assessments of subjective urinary symptoms and quality of life indicated within-participant improvements across all standardized questionnaires (Fig 3). Total scores for the UDI-6, IIQ-7, and OABSS decreased significantly post-treatment. These improvements were sustained through the follow-up period, indicating that the relief of LUTS and their negative impact on daily life was maintained at the three-month follow-up.

**Fig 4:**
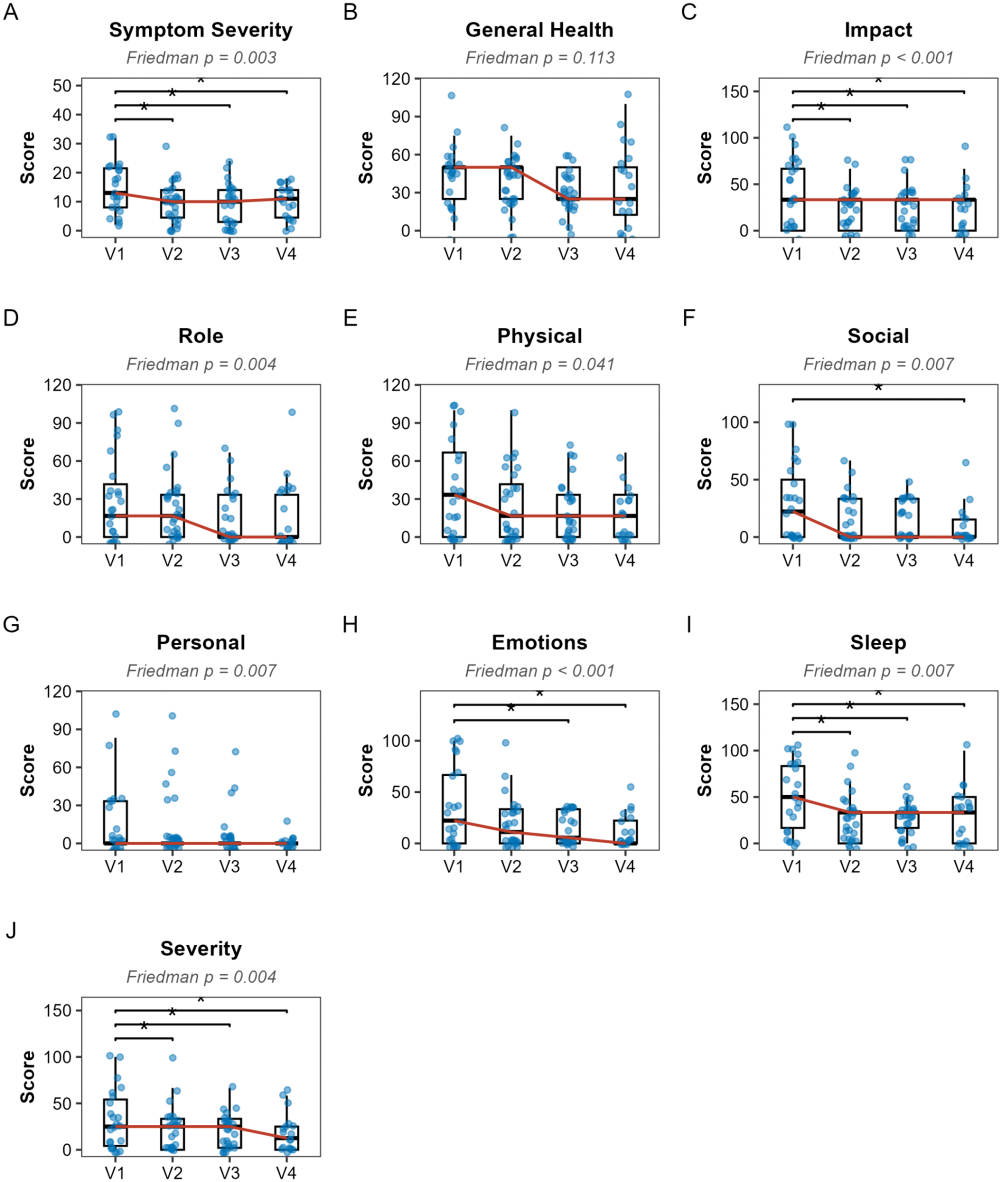
Quality of life assessment across multiple domains of the King’s health questionnaire (KHQ). Panels A–J represent **(A)** Symptom Severity Scale, **(B)** General Health Perception, **(C)** Incontinence Impact, **(D)** Role Limitations, **(E)** Physical Limitations, **(F)** Social Limitations, **(G)** Personal Relationships, **(H)** Emotions, **(I)** Sleep/Energy, and **(J)** Severity Measures. Scores range from 0 to 30 for the Symptom Severity Scale and 0 to 100 for all other domains. Significant reductions in scores signify improved quality of life. *p < 0.05, **p < 0.01, ***p < 0.001 compared to baseline (V1). (The Friedman test was used for global repeated-measures comparisons. When significant, post-hoc pairwise comparisons with baseline were performed using Holm–Bonferroni correction.).

In the pelvic floor-specific quality of life assessment (Fig 4), significant reductions were observed in many domains of the KHQ, including the symptom severity scale, incontinence impact, sleep, emotions, as well as social limitations.

### Female sexual function

**Fig 5:**
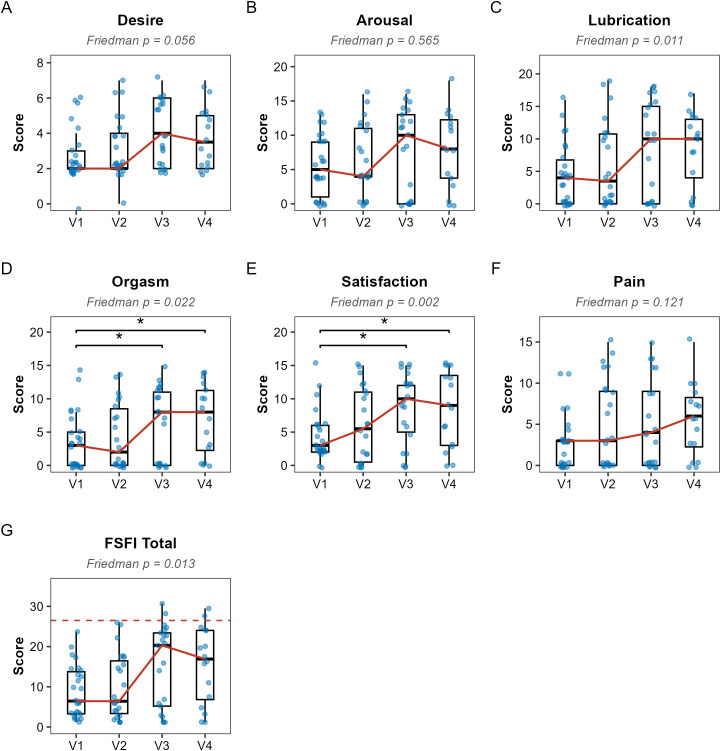
Impact of photobiomodulation on female sexual function domains (FSFI). Assessment included **(A)** Desire, **(B)** Arousal, **(C)** Lubrication, **(D)** Orgasm, **(E)** Satisfaction, **(F)** Pain, and **(G)** FSFI Total Score. The dotted red line in Panel G denotes the clinical cut-off value of 26.5 for sexual dysfunction. The analysis only included participants reporting sexual activity at each time point (n = 22, 20, and 15 for V2, V3, and V4, respectively). While orgasm and satisfaction showed robust significance in both global and post-hoc tests, the FSFI total score (*p* = 0.013) and lubrication (*p* = 0.011) showed significance only in the global Friedman test. *p < 0.05, **p < 0.01, ***p < 0.001 compared to baseline (V1).

For sexual function analysis, only sexually active participants were included, with the number of evaluable cases being n = 22 at V2, n = 20 at V3, and n = 15 at V4. Regarding sexual function, PBM therapy demonstrated a positive impact on the overall quality of sexual life (Fig 5). A significant global improvement was observed in the FSFI total scores (Friedman *p* = 0.013, panel G) and the lubrication domain (Friedman *p* = 0.011, panel C). However, despite these significant global trends, specific pairwise comparisons between baseline and subsequent timepoints for these two metrics did not reach statistical significance after applying the conservative Holm–Bonferroni correction. Furthermore, only two participants reached the clinical cut-off value (≥ 26.5) at the immediate post-treatment assessment and the three-month follow-up, respectively.

On the other hand, the orgasm and overall satisfaction domains demonstrated significant improvements in both the global Friedman test and subsequent post-hoc pairwise comparisons (*p* < 0.05). Other sub-indices, including desire (*p* = 0.06), arousal (*p* = 0.56), and sexual pain (*p* = 0.12), exhibited gradual upward trends in median scores but remained statistically non-significant.

### Clinical improvement rates

**Fig 6:**
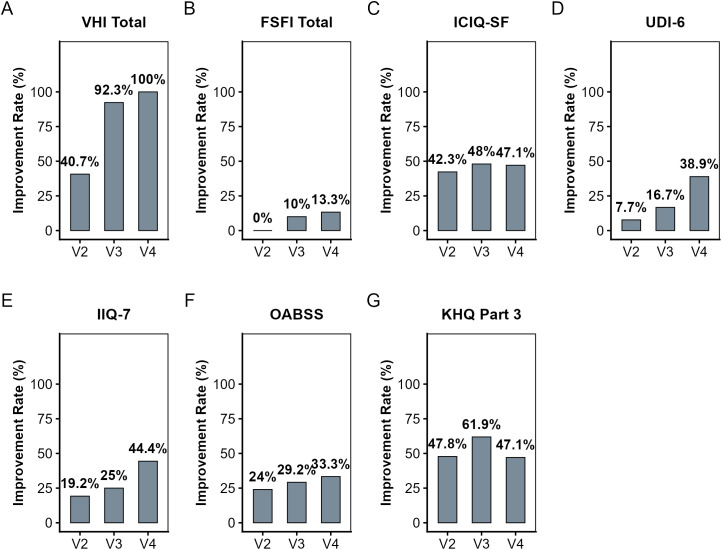
Clinical improvement rates of photobiomodulation at multiple follow-up intervals. Clinical improvement rate was defined by established clinical thresholds: VHI > 15; FSFI ≥26.5; minimal clinically important differences (MCID) of 1.7 for ICIQ-SF, 3 for OABSS, and 5 for KHQ domains; and minimal detectable change (MDC) of 4.1 for UDI-6 and 3.8 for IIQ-7. Individual panels represent: **(A)** VHI total score, **(B)** FSFI total score, **(C)** ICIQ-SF, **(D)** UDI-6, **(E)** IIQ-7, **(F)** OABSS Total Score, and **(G)** KHQ symptom severity scale. Data labels indicate the percentage of participants showing clinical improvement at each visit. V2, pre-4^th^ treatment; V3, pre-8^th^ treatment; V4, 3-month follow-up.

To further evaluate the clinical utility of PBM, clinical improvement rate was assessed using established MCID, MDC, and clinical cutoff thresholds (Fig 6). The objective vaginal health parameters showed the most obvious response; the proportion of participants achieving the non-atrophic status cutoff (>15) reached 40.7% at V2, 92.3% at V3, and 100% at V4 (panel A). For sexual function, although significant score improvements were observed, the proportion of participants reaching the diagnostic cutoff (≥ 26.5) remained limited at V4 (panel B). For urinary-related distress and symptom severity (panels C–G), a subset of patients derived clinically meaningful benefits, though achievement rates for these parameters generally remained below 50% throughout the follow-up period except for KHQ symptom severity at V3.

## Discussion

The current single-center prospective study evaluated PBM for GSM primarily through serial VHI assessment, with secondary evaluation of urinary and sexual symptom outcomes. The findings suggest that PBM may improve both objective vaginal health and subjective symptoms, with changes appearing shortly after treatment initiation and remaining evident at the three-month follow-up. Notably, all participants exhibited measurable improvement in VHI scores, highlighting a consistent clinical response to this non-hormonal intervention.

Over the past two decades, the utilization of vaginal laser therapy has increased, predominantly involving ablative CO_2_ lasers and non-ablative Er:YAG lasers [25,26]. More recently, non-ablative dual-wavelength diode laser therapy has also been reported as a potential treatment for GSM, with improvements in vaginal health, GSM symptoms, and sexual function observed in a single-center prospective study [27]. These energy-based devices primarily function through tissue heating or photothermal stimulation to trigger tissue remodeling. While previous studies have acknowledged the short-term effects of energy-based devices for GSM and SUI, clinical practice reveals certain practical limitations. Minor adverse effects, such as heat sensations and tenderness during the procedure, are occasionally reported [28]. Furthermore, the procedural requirement to manipulate a relatively thick probe—often without lubricant to avoid interference with the laser beam—can cause rubbing pain. This is particularly problematic for patients with GSM and low VHI scores, which often indicate severe mucosal atrophy and heightened tissue fragility.

Given that participants with severe GSM symptoms frequently exhibit extremely poor vaginal health at baseline, the PBM protocol provides a critical clinical advantage. Photobiomodulation appears to offer a different profile regarding patient comfort compared to high-energy devices. In the cohort, the therapy was generally perceived as comfortable. No participants withdrew due to procedural discomfort, and no adverse effects were observed. The PBM protocol utilizes a static silicone probe that does not require rotation or friction, which may be beneficial for fragile or highly symptomatic patients [11]. Moreover, the design of the PBM probe facilitates consistent energy delivery even in narrowed vaginal canals. The absence of observed adverse effects in this study may have contributed to the high compliance rate.

While GSM is histologically characterized by epithelial thinning, reduced vascularization, and compromised extracellular matrix (ECM) integrity [29], the clinical improvements observed in GSM symptoms following AlGaInP diode laser (660 nm) therapy may be biologically supported by its effects on cellular bioenergetics and tissue remodeling. According to the mechanisms described by Hamblin et al. [30,31], at the primary cellular level, mitochondria in stressed or damaged cells—such as atrophic vaginal epithelial cells—produce excess nitric oxide (NO). This NO binds to cytochrome c oxidase (CCO), inhibiting the electron transport chain and decreasing ATP synthesis. Photons in the 620–700 nm range are absorbed by the CCO (mitochondrial respiratory chain Complex IV), facilitating the photodissociation of inhibitory NO. This process restores the electron transport chain, increases mitochondrial membrane potential, and enhances ATP production. Furthermore, 660 nm laser irradiation of human skin fibroblasts has been shown to modulate the expression of genes involved in collagen production, cellular adhesion, remodeling, cytoskeletal organization, and signaling cascades involving growth factors and inflammatory cytokines [32]. These primary effectors act as crucial second messengers that activate key transcription factors and signaling cascades, most notably the HIF-1α, NF-κB, and TGF-β/Smad pathways [33,34]. This activation stimulates local fibroblast proliferation and promotes the synthesis of new collagen and ECM, thereby restoring the structural elasticity of the vaginal wall. Moreover, PBM-induced upregulation of HIF-1α and VEGF promotes angiogenesis [33], which may restore the diminished mucosal blood flow characteristic of GSM. Concurrently, PBM exerts localized anti-inflammatory effects by downregulating pro-inflammatory cytokines such as TNF-α and IL-6, facilitating optimal epithelial regeneration [9,35].

While PBM is established in wound healing and pain management [36,37], its application as a non-hormonal regenerative treatment for GSM remains a novel approach. By synergistically enhancing fibroblast activity, neovascularization, and epithelial proliferation, PBM might facilitate a cumulative reparative process that restores a favorable vaginal microenvironment [38]. This is evidenced by improved lubrication, stabilized pH, and enhanced tissue elasticity, ultimately resulting in the progressive increase of VHI scores observed from V2 through V4.

An intriguing observation in this study is the discrepancy between objective anatomical recovery and subjective functional attainment in sexual health. Despite the significant and substantial improvement in vaginal health, only a small number of participants reached the clinical cut-off for functional recovery (FSFI ≥26.5). This could suggest a temporal lag between physiological repair and psychological restoration.

The recovery of sexual function remains a multifactorial process. In certain Asian contexts, sexual health issues in postmenopausal women may be understated or stigmatized, leading to internalized perceptions of dysfunction that may not resolve immediately upon physiological healing [39]. Theoretically, the reduction of dyspareunia and the restoration of lubrication are necessary first steps; however, the subsequent recovery of desire and arousal might require extended follow-up or multidisciplinary support. Even though a general improvement was observed in sexual function, the trend was likely offset by the reduced sample size at later follow-up stages in the post-hoc analysis. The above findings may indicate that three months is a minimum threshold to observe the beginning of this transition, and the definition of “normal” function in a geriatric population may benefit from age-specific adjustments rather than a universal numerical threshold (FSFI ≥26.5) [20].

In an aging population, the management of GSM must account for the complexities of geriatric care. While local hormonal therapy is efficacious, it may be non-applicable for survivors of gynecological malignancies or practically challenging for some elderly individuals to apply consistently. Furthermore, GSM frequently coexists with LUTS, which typically necessitate oral pharmacological interventions. The systemic side effects of such medications can sometimes lead to poor compliance or adverse drug interactions in elderly patients.

The present study shows that PBM was associated with alleviated LUTS across multiple scales (UDI-6, IIQ-7, and OABSS). This synergistic efficacy may suggest the role of PBM as an integrated therapeutic modality for GSM patients with comorbid urinary symptoms [11]. By addressing both vaginal and urinary distress through a minimally invasive intervention, PBM could potentially offer a pathway to mitigate the clinical complexities associated with polypharmacy.

Despite encouraging results, this study has several limitations. First, while the present study was designed as a single-arm prospective pilot study to establish preliminary clinical outcomes, tolerability, and reproducible radiometric parameters for this emerging application in GSM, the absence of a control group remains the major limitation. Without a placebo or active comparator group, causal inference is limited, and the observed improvements cannot be definitively distinguished from placebo responses or natural symptom variability. Therefore, the present findings should be interpreted as preliminary and hypothesis-generating. A subsequent controlled study is currently being organized to further validate these findings. Second, the small sample size may have limited the statistical power for certain secondary outcomes following conservative Friedman and Holm–Bonferroni corrections. To mitigate the limitations of relying on statistical significance alone, clinical improvement was evaluated using MCID, MDC, and cutoff scores to support clinically meaningful interpretation. However, these threshold-based analyses do not replace the need for controlled validation. Future randomized placebo-controlled trials with larger sample sizes and longer follow-up are needed to confirm efficacy, durability, and comparative effectiveness.

## Conclusion

Photobiomodulation appears to provide a comfortable, economical, and adaptable therapeutic option for women with GSM, showing improvements in both objective vaginal health and subjective quality of life. Its favorable tolerability profile may offer a potential alternative for patients who are unsuitable for hormonal therapy or find traditional high-energy laser procedures difficult to tolerate. Furthermore, PBM could serve as an integrated therapeutic modality by reducing the clinical complexities and risks associated with polypharmacy for LUTS in the geriatric population. As a novel application in this field, PBM demonstrates a promising safety and efficacy profile that warrants further clinical investigation.

## Supporting information

S1 File STROBE checklist. (PDF)
